# The Role of Childhood Obesity in Early-Onset Type 2 Diabetes Mellitus: A Scoping Review

**DOI:** 10.7759/cureus.48037

**Published:** 2023-10-31

**Authors:** Uchechukwu S Oranika, Oluwakemi L Adeola, Thelma O Egbuchua, Okelue E Okobi, Daad G Alrowaili, Ayokunle Kajero, Oluwagbemiga M Koleowo, Emeka Okobi, Ademiluyi B David, Jennifer C Ezeamii

**Affiliations:** 1 Family Medicine, University of Alberta Hospital, Edmonton, CAN; 2 Nutritional Sciences, Howard University, Washington DC, USA; 3 Pediatrics and Neonatology, Delta State University Teaching Hospital, Oghara, NGA; 4 Family Medicine, Larkin Community Hospital Palm Springs Campus, Hialeah, USA; 5 Family Medicine, Medficient Health Systems, Laurel, USA; 6 Family Medicine, Lakeside Medical Center, Belle Glade, USA; 7 Human Genetics, Howard University, Washington DC, USA; 8 Sexual and Reproductive Health/Adolescent Health, Ondo State Primary Health Care Development Agency, Akure, NGA; 9 Medicine, College of Medicine, University of Lagos, Lagos, NGA; 10 Dentistry, Ahmadu Bello University Teaching Hospital, Zaria, NGA; 11 Medical Laboratory Sciences, Asokoro General Hospital, Abuja, NGA; 12 Nursing, University of Nigeria, Nsukka, NGA

**Keywords:** global impact, comorbidities, obesity determinants, public health, risk factors, prevalence, early-onset type 2 diabetes mellitus, childhood obesity

## Abstract

Childhood obesity is a growing concern worldwide, with significant implications for public health. Of particular interest is its association with the early onset of type 2 diabetes mellitus in children. Exploring this relationship and identifying contributing factors may help strengthen understanding of this complex relationship. Factors such as family history, gender, and sedentary lifestyle, and poor dietary habits, insulin resistance in adipose tissue have been identified as significant contributors to early-onset type 2 diabetes. The rising prevalence of childhood obesity and its association with diabetes necessitates early interventions to mitigate this concerning trend and ensure a lasting impact on public health.

This scoping review aims to investigate the relationship between childhood obesity and early-onset type 2 diabetes mellitus while also exploring other contributing factors. We employed a standardized framework for reviews to analyze relevant literature published in English between 2000 and 2021. Only primary research, systematic reviews, and meta-analyses addressing the association between childhood obesity and early-onset type 2 diabetes mellitus were included. The review adhered to the Preferred Reporting Items for Systematic Reviews and Meta-Analyses (PRISMA) format. Out of the 3614 articles assessed, 17 were ultimately incorporated into this scoping review as they met the inclusion criteria.

The majority of the literature primarily represented North American studies, with no inclusion of studies from South America. The findings from these studies have highlighted several factors contributing to type 2 diabetes mellitus development. Most of the studies associated obesity development with hypertension and unfavorable lipid profiles. It is important to acknowledge that these findings are derived from the available literature and may not encompass the entirety of research on childhood obesity and early-onset type 2 diabetes mellitus. Furthermore, the limited regional diversity in the selected studies may restrict the generalizability of these findings to other geographic regions. Additional research involving diverse populations is imperative for a comprehensive understanding of the link between childhood obesity and early-onset type 2 diabetes mellitus. Insulin resistance in adipose tissue among obese individuals contributes to the transition from obesity to type 2 diabetes mellitus. Notably, this transition occurs at approximately half the duration in obese youths compared to obese adults, where it typically takes around a decade.

The increasing prevalence of childhood obesity and diabetes mellitus in high-income, low-income, and middle-income countries necessitate collective efforts to reduce the increasing rates of early-onset type 2 diabetes in children. This scoping review, therefore, seeks to underscore the importance of early interventions with regard to ensuring a lasting impact capable of extending into adulthood.

## Introduction and background

According to the World Health Organization (WHO), obesity is characterized by the abnormal accumulation of body fat, leading to impaired physiological functions and adverse health outcomes. It is quantitatively defined as having a body mass index (BMI) exceeding 30, providing an objective measure for clinical diagnosis. The prevalence of childhood obesity has been steadily increasing in the twenty-first century, posing a significant public health challenge [1]. The projected impact of childhood obesity is alarming, with nearly half of the world's children expected to be affected by 2020, especially in regions where obesity has overtaken underweight as a primary health concern [2].

The determinants of excessive weight gain in children mirror those observed in adults, as outlined by the Centers for Disease Control and Prevention [3]. Obesity is a complex condition influenced by various factors, including genetic predisposition, environmental influences, dietary patterns, cultural factors, metabolic processes, individual behaviors, and family history [4]. Furthermore, the increasing prevalence of childhood diabetes mellitus has been linked to a rise in comorbidities such as type 2 diabetes mellitus, hypertension, asthma, dental issues, and liver disorders [5].

Early-onset type 2 diabetes mellitus is a significant public health concern. Previously more common in adults, type 2 diabetes mellitus is now increasingly prevalent among younger age groups, including children, adolescents, and young adults [6]. While there's no universally agreed-upon upper age limit for classifying early-onset diabetes mellitus, it typically refers to diabetes mellitus occurring before age 40. What's concerning about early-onset type 2 diabetes mellitus is mounting evidence suggesting its more aggressive nature compared to later-onset type 2 diabetes mellitus, characterized by a quicker onset and progression of complications affecting both large and small blood vessels [7]. The estimated global prevalence of early-onset type 2 diabetes mellitus is 371 million individuals, including children, and is projected to reach 552 million by 2023 [8].

Several conditions and risk factors are associated with early-onset type 2 diabetes mellitus, including age, obesity or overweight, family history, lifestyle, race or ethnic origin, hypertension, high cholesterol levels, malnutrition, and autoimmune, genetic, and environmental factors [9]. Numerous studies have shown a clear link between childhood obesity and the development of type 2 diabetes mellitus in children [10]. One study reported that obese children are four times more likely to develop type 2 diabetes mellitus compared to those with normal weight [11].

Given that childhood obesity often leads to additional complications such as early-onset type 2 diabetes mellitus [12], this study aims to investigate the role of childhood obesity in the development of early-onset diabetes mellitus. The specific objectives include determining the prevalence of childhood obesity and early-onset type 2 diabetes mellitus, analyzing the distribution patterns of these conditions, identifying factors contributing to childhood obesity and early-onset type 2 diabetes mellitus, exploring current knowledge, attitudes, and practices related to these issues, and identifying gaps in the understanding and management of childhood obesity and early-onset type 2 diabetes mellitus.

## Review

Methodology

This review employed the Arksey and O'Malley review methodology, which comprises five stages for comprehensively mapping existing literature [13]. The initial stage involved formulating research questions, which were derived from the introductory section of this review. In the subsequent stage, relevant literature was identified by searching databases such as PubMed, NCBI, Science Direct, and Springer Link. Various keywords, including "obesity," "childhood obesity," "onset diabetes mellitus," "health institutions," "early-onset type 2 diabetes mellitus," "factors contributing to obesity and early-onset type 2 diabetes mellitus," "the association between childhood obesity and onset diabetes mellitus," and "identifying research gaps in childhood obesity studies," were used to refine the search results. The literature search was restricted to materials published between 2001 and 2021, focusing on up-to-date research on childhood diabetes mellitus and early-onset diabetes mellitus. Only primary research, systematic reviews, and meta-analyses conducted in English and addressing the relationships between childhood obesity and early-onset diabetes mellitus were included in the review.

**Figure 1:**
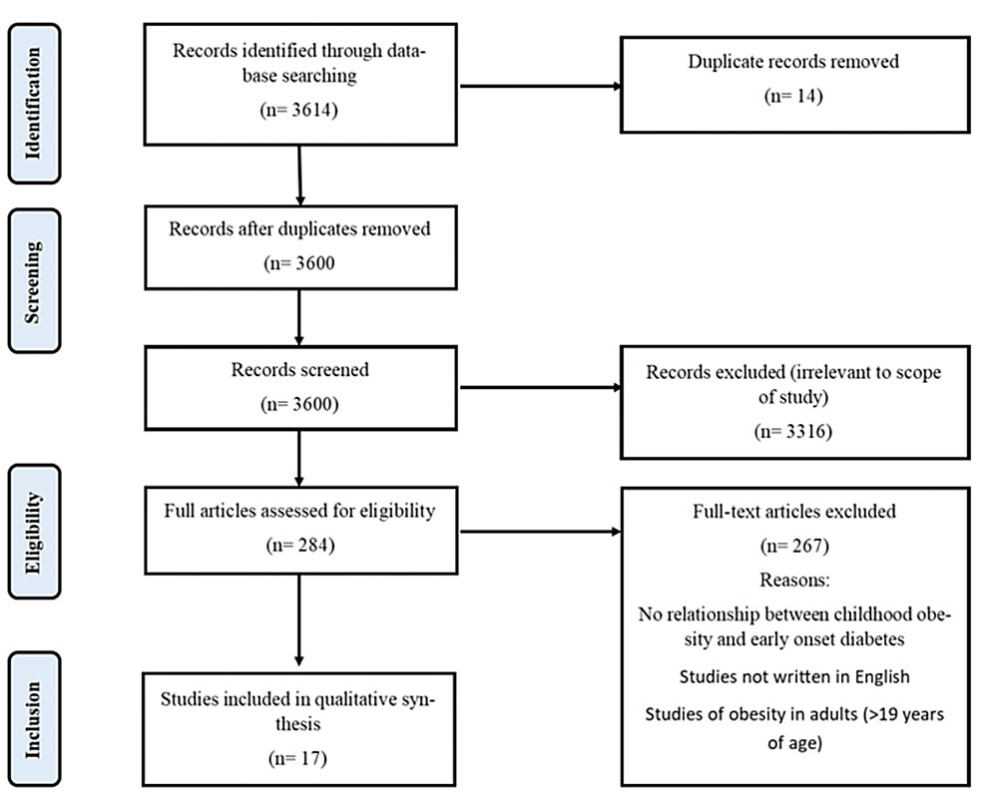
PRISMA flow diagram of selected studies PRISMA: Preferred Reporting Items for Systematic Reviews and Meta-Analyses

Following the initial compilation of literature, duplicate entries were removed (as depicted in the PRISMA Figure 1 below) [14-46]. Subsequently, each piece of literature underwent title and abstract screening, guided by predefined inclusion criteria, to select studies for full-text review. The studies chosen for full-text review were subject to further evaluation to determine their final inclusion.

Data extracted from the selected literature pieces were then categorized into eight sections: authors, publication year, study location, research design, characteristics of the study population, research objectives, conclusions, and interventions. The findings from data mapping were subsequently summarized, synthesized, and reported using various analytical methods, including the presentation of results in frequency and/or percentage. As depicted in Table 1, the characteristics of the included studies are summarized, highlighting key details such as study design and publication year. 

**Table 1:** Table of study characteristics LGA, local government area; T1DM, type 1 diabetes mellitus; T2DM, type 2 diabetes mellitus; MDM, monogenic diabetes mellitus; NGT, normal glucose tolerance; IGT, impaired glucose tolerance; OGTT, oral glucose tolerance test; HbA1c, glycosylated hemoglobin; AIRg, acute insulin response to glucose; PI, proinsulin; DI; diabetes insipidus; CHD, coronary heart disease; BMI, body mass index

Author	Year	LGA/stat e/country	Research design	Population characteristics	Objective of the study	Conclusions
Kumar et al. [25]	2021	India	Cohort study	17,865 adolescent boys and 17,965 adolescent girls	To assess the prevalence of pre-diabetes mellitus/T2DM among adolescents (10-19 years) and its association with different measures of overweight/obesity in India	The high prevalence of pre-diabetes mellitus and diabetes mellitus among adolescents portrayed serious public health concerns in India. Body mass index and subscapular skinfold thickness were positively associated with pre-diabetes mellitus/diabetes mellitus conditions among adolescents.
Oluwayemi et al. [10]	2021	Nigeria	Cross-sectional study	21 children managed for childhood obesity at the Ekiti State University Teaching Hospital (EKSUTH), South West, Nigeria for 10 years from August 2010 to August 2020	To determine the prevalence of obesity and the associated morbidities seen at the pediatric endocrinology clinic of EKSUTH	Obesity is a common disease condition among children attending pediatric endocrine clinics. Strategies to control obesity and the progression of severity of obesity may have a place in reducing the prevalence of hypertension in obese children and adolescents.
Twig et al. [15]	2020	Israel	Cohort study	1,462,362 adolescents (59% men, mean age 17.4 years) during 1996-2016	To assess the association of adolescent obesity with incident T2DM in early adulthood	Severe obesity significantly increases the risk of T2DM in early adulthood in both sexes. The rise in adolescent severe obesity will likely increase diabetes mellitus incidence in young adults in the coming decades.
Simchoni et al. [14]	2020	Israel	Cohort study	121,490 normoglycemic adolescents (range 16-20 years old), including 93,806 native Israelis 3rd generation in Israel) and 27 684 Israelis of Ethiopian origin	To assess the association between adolescent BMI and early-onset (<40 years) T2DM among Israelis of Ethiopian origin	Ethiopian origin is a risk factor for early-onset T2DM among young men at any BMI.
Kim et al. [19]	2019	United States of America	Cross-sectional study	205 adolescents	To investigate adipose insulin resistance index in youth across the spectrum of adiposity from normal weight to obese and the spectrum from normal glucose tolerance to impaired glucose tolerance to T2DM	Adipose-1R is a simple surrogate estimate that reflects pathophysiological alterations in adipose tissue insulin sensitivity in youth, with progressive deterioration from normal weight to obese and from NGT to IGT to T2DM.
Candler et al. [41]	2018	United Kingdom	Cross-sectional study	106 cases of T2DM diagnosed in children aged <17 years between April 1, 2015, and April 30, 2016	To estimate the incidence of T2DM in children aged <17 years, compare this with similar data 10 years ago, and characterize clinical features at diagnosis in the United Kingdom and the Republic of Ireland	T2DM remains far less common than T1DM in childhood in the United Kingdom. Still, cases continue to rise, with significantly increased incidence among girls and South-Asian children over a decade. Female gender, family history, non-white ethnicity, and obesity were strongly associated with the condition.
Chedjou- Nono et al. [16]	2017	Cameroon	Cross-sectional study	Children aged 3 to 17 years who were being followed up for obesity at the pediatric endocrinology unit of the Mother and Child Center of the Chantal BIYA Foundation in Yaounde, Cameroon		Obesity in children is associated with early-onset metabolic disorders such as dyslipidemia, high blood pressure, and T2DM. The screening and management of these complications is therefore recommended.
Wheelock et al. [42]	2016	United States of America	Prospective cohort study	5532 non-diabetic Pima Indian children 5-19 years old	To determine metabolic risk factors and T2DM incidence in American Indian children	BMI and impaired glucose tolerance in children serve as strong predictors of T2DM, while other components of the "metabolic syndrome" do not exhibit the same predictive strength.
Zabeen et al. [43]	2016	Bangladesh	Cross-sectional study	77 children and adolescents diagnosed <18 years who had features of T2DM	To describe the baseline characteristics of children and adolescents diagnosed <18 years who had features of T2DM	T2DM is emerging as a problem in children and adolescents in Bangladesh.
Al Amiri et al. [44]	2015	United Arab Emirates	Cross-sectional study	Overweight/obese Emirati students; grades 6-12 (age 11-17 years) from 16 government schools in Sharjah, United Arab Emirates	To estimate the prevalence of pre-diabetes mellitus and T2DM among overweight/obese children and adolescents using different diagnostic/screening methods in comparison	The prevalence of pre-diabetes mellitus and diabetes mellitus was high among overweight/obese Emirati children and adolescents. The numbers for pre-diabetes mellitus were considerably higher when using HbAlc compared to OGTT. Overall adiposity, family history of T2DM, employment, and high levels of triglycerides were risk factors associated with abnormal glycemic testing.
Elder et al. [21]	2015		Cohort study	41 obese adolescents with normal glucose tolerance were studied longitudinally over 4 years with serial measures of the acute insulin response to glucose (AIRg) as well as proinsulin (PI) concentrations	To identify pathophysiologic changes that lead to the onset of T2DM in adolescents	Conversion from normal glucose tolerance to T2DM in adolescents can occur rapidly, and the onset of T2DM is heralded by a substantial decrease in AIRg and DI and increased release of PI. These results support loss of cell function as the proximate step in developing T2DM in this age group.
Park et al. [45]	2013	Britain	Cohort study	11,477 individuals born in 1946, 1958 and 1970	To assess whether being overweight and obese in childhood and adolescence contributes to excess cardiovascular risk in adults	T2DM and, to a lesser extent, CHD risk may be affected by being overweight at all stages of life. In contrast, hypertension risk is associated more strongly with weight status in adulthood.
Osman et al. [18]	2013	Sudan	Cross-sectional study	985 children attending the clinic at Jabir Abu Izz Diabetic Center from January 2006 to December 2009	To determine the prevalence of T2DM among children and adolescents	The study confirms that T2DM is emerging as a health problem among children and adolescents in Sudan mostly as a result of obesity, particularly among high socioeconomic groups in urban areas and more prevalent in certain ethnic groups
Keller et al. [23]	2012	United States of America	Cross-sectional study	227 African American and 112 Hispanic American pediatric patients were diagnosed with T1DM or T2DM	To test the hypothesis that clinical observations made at patient presentation can distinguish T2DM from T1DM in pediatric patients aged 2 to 18	The distinction of T2DM from T1DM at patient presentation was possible with good sensitivity and specificity using only three easily assessed variables: age, gender, and BMI z-score. In African-American pediatric diabetes mellitus patients, gender was the strongest predictor of T2DM, while BMI z-score was the strongest predictor in Hispanic patients. This suggests that race/ethnic-specific models may be useful to optimize the distinction of T1DM from T2DM at presentation.
Lipton et al. [22]	2011	United States of America	Cross-sectional study	111 patients aged 0- 17	To explore whether it is possible to predict a child's eventual diabetes mellitus phenotype using characteristics at the initial presentation	Ten percent of subjects had MDM, and 15% had T2DM at 8 years. Although no onset feature was reliable, ketoacidosis and hyperglycemia were more likely to predict T1DM; obesity and African-American ethnicity made T2DM more likely. At diagnosis, features of T2DM, in addition to obesity, were strongly predictive of eventual T2DM phenotype. Given the significant percentage who changed or had mixed phenotypes, carefully tracking all young people with diabetes mellitus is essential to determine the eventual disease type correctly.
Al Mamun et al. [24]	2009	Australia	Cohort study	2,639 young adults from the Mater- University study of pregnancy (MUSP) and its outcomes	To examine the prospective association of childhood BMI z-score and BMI categories (normal or overweight) with young adult diabetes mellitus	Overweight and increasing BMI z-score in childhood is an independent predictor of young adults' T1DM and T2DM. Childhood BMI may be central to the development and rising incidence of all diabetes mellitus.
Drake [46]	2001	England	Cross-sectional study	36 children with severe obesity of prepubertal onset	To investigate pancreatic function in children attending an obesity clinic.	Metabolic abnormalities predictive of type II diabetes mellitus occur in severely obese white children.

Results**

Various studies have reported varying prevalence rates of obesity among children and adolescents diagnosed with type 2 diabetes mellitus across different regions. For instance, in a Bangladeshi study [43], the prevalence was 58.4%, whereas a European study documented a prevalence of 81.1% [6]. Among mixed populations comprising both non-diabetic and diabetic participants, the prevalence of obesity ranged from 0.15% in an Israeli study [14] to 6.1% in another Israeli study [15]. Table 2 provides a comprehensive summary of the included studies, including key characteristics, baseline characteristics, and relevant outcomes.

**Table 2:** Key characteristics and outcomes of included studies T2DM, type 2 diabetes mellitus; FBG, fasting blood glucose; 2HPP, 2-hour postprandial blood sugar; RBG, random blood glucose; HbA1c, glycosylated hemoglobin; OGTT, oral glucose tolerance test; a-c & e, percentage with childhood overweight/obesity; d and f, type 1 and 2 diabetes mellitus respectively

SN.	Author	Age range	Location	Population size	Mean/median age	Male: Female ratio	Percentage of childhood obesity (%)	Percentage with onset T2DM	Test
1	Kumar et al. [25]	10-19 years	India	35,830	NA	1:1	4.4^a^	8.4	HbA1c
2	Oluwayemi et al. [10]	1-16 years	Nigeria	21	8.8	1:2	100	4.8%	FBG
3	Twig et al [15]	16-19 years	Israel	1,462,362	17.3	1.4:1	6.1%	0.14	Medical record
4	Simchoni et al. [14]	16-20 years	Israel	121,490	NA	1.8:1	0.15^b^	0.25	HbA1c/RBG
5	Kim et al. [19]	10-19 years	United States of America	205	NA	1:1.9	36.1	14.1	HbA1c/2HPP
6	Candler et al. [41]	<17 years	United Kingdom	106	14.3	1:2	81.1	100	Medical record
7	Chedjou- Nono et al. [16]	3-17 years	Cameroon	38	9.9	1.2:1	100	2.6	FBG
8	Wheelock et al. [42]	5-19 years	United States of America	5,532	11.4	1:1.1	59.8%^c^	23.2%^d^	2HPP
9	Zabeen et al. [43]	≤18 years	Bangladesh	77	NA	1:1.8	58.4	100	FBG/2HPP
10	Al Amiri et al. [44]	11-17 years	United Arab Emirates	1,034	14.7	1.2:1	77.7	0.87	OGTT
11	Elder et al. [21]	NA	United States of America	36	NA	1:3	100	11.1	FBS
12	Park et al. [45]		Britain	11,447		1:1	1.0	2.0	Self-reported
13	Osman et al. [18]	<19 years	Sudan	958	NA	1:1.2	55.3^e^	4.0	
14	Keller et al. [23]	2-18 years	United States of America	339		1:1.2	57.2	41.0^f^	HbA1c
15	Lipton et al. [22]	0-17 years	United States of America	111	16.1	1:1.5	16.2	15.3	Medical record
16	Al Mamun et al. [24]	5 years	Australia	2,639	5.0	NA	3	1.55	Self-reported
17	Drake [46]	≤18 years	England	36	10.4	1:1.1	100%	2.88%	FBG/2HPP

Similarly, the prevalence rates of type 2 diabetes mellitus among obese children exhibit regional variations. Studies conducted in Africa, specifically in Nigeria and Cameroon, reported prevalence rates of 4.8% and 2.6%, respectively [10,16]. In contrast, a Sudanese study reported a prevalence of 4.0% for type 2 diabetes among a combined population of normal-weight, overweight, and obese children and adolescents [18].

Studies focusing on young obese populations in the United States reported significantly higher prevalence, ranging from 11.1% to 41.1% [19-23]. In contrast, Israeli studies reported the lowest prevalence of type 2 diabetes mellitus, with rates of 0.14% and 0.25% [14,15], followed by an Australian study reporting a prevalence of 1.55% [24]. These variations underscore the regional disparities in the burden of type 2 diabetes mellitus among obese children, emphasizing the need to consider the context and population characteristics of each study when interpreting and comparing findings.

The reviewed literature included children and adolescents aged one to 19, with one Israeli study extending the upper age limit to 20 [14]. Population sizes varied widely, ranging from 21 participants in a Nigerian study [10] to 1,462,362 participants in an Israeli study [15]. In most of the literature, female participants outnumbered males, with the most significant gender imbalance observed in an American study, where the male-to-female ratio was 1:3 [21].

Numerous risk factors associated with the development of type 2 diabetes mellitus were investigated in several studies. Lipton et al. reported that a family history of diabetes mellitus and obesity significantly increased the risk of developing type 2 diabetes mellitus in later life [22]. Kumar et al. found a significant association between increased BMI and subscapular skinfold thickness with diabetes mellitus [25]. Gender was identified as a risk factor in the study by Kumar et al., with adolescent boys being more susceptible to diabetes mellitus than girls [25]. A sedentary lifestyle, reduced physical activity, and poor dietary habits, including the consumption of junk food, sweetened foods, and high-fat diets, were also reported as factors increasing the risk of diabetes mellitus development.

Early detection of diabetes mellitus, or even pre-diabetes mellitus, carries a lower long-term risk than remaining undiagnosed [25]. Kim et al. revealed a correlation between obesity and an unfavorable lipid profile, with obese individuals showing significantly higher LDL, VLDL, triglycerides (TGs), total cholesterol, and lower levels of HDL [19]. Childhood obesity also contributes to comorbidities such as hypertension, dyslipidemia, dysglycemia, and acanthosis nigricans compared to their counterparts of similar age and sex [16].

Furthermore, Kim et al. found that obese youth with impaired glucose tolerance exhibited significantly higher levels of free fatty acids than their normal-weight counterparts, despite having up to three times the fasting insulin levels [19]. The study also reported an increase in adipose tissue insulin resistance from lean to obese individuals and from individuals with normal glucose tolerance to impaired glucose tolerance and type 2 diabetes mellitus. Another study by Elder et al., which included 41 obese adolescents, observed that the progression from a normal glycemic state in non-diabetic obese individuals to type 2 diabetes mellitus could occur in as little as 1 to 4 years [21].

Additionally, the study conducted by Al Amiri et al. revealed that when used alone, HbA1c may have lower specificity compared to oral glucose tolerance tests in accurately diagnosing diabetes mellitus [44]. In another investigation by Lipton et al., the diagnosis of type 1 diabetes mellitus relied on assaying for glutamic acid decarboxylase (GAD) antibody and insulin auto-antibody (IAA/IA2), while C-peptide levels were used to establish type 2 diabetes mellitus [22]. The measurement of C-peptide levels can indicate beta cell mass depletion, with residual beta cell function typically preserved in type 2 diabetes mellitus. Consequently, patients with type 2 diabetes mellitus can effectively manage their condition through oral hypoglycemic drugs and dietary plans. These findings underscore the importance of employing multiple diagnostic methods and considering the specific characteristics of each type of diabetes mellitus to ensure accurate diagnosis and appropriate management strategies.

Discussion

The literature concerning childhood obesity and early-onset diabetes mellitus predominantly originates from high-income countries, with North America and Asia contributing most of the studies. European studies are also notable, while African and Australian studies are comparatively limited. This disparity highlights a research gap regarding the association between childhood obesity and early-onset diabetes mellitus, especially in low and middle-income countries, and underscores the need for more comprehensive global representation.

The majority of studies reviewed fall into the categories of cross-sectional or cohort studies, offering valuable insights into the prevalence and connections between obesity and early-onset diabetes mellitus within specific populations. For instance, various studies have reported an obesity prevalence of 16.2% among the American population below 17 years of age [22,26], which aligns with the Centers for Disease Control and Prevention report indicating an obesity prevalence of approximately 19.7% in children aged 2 to 19 years [27]. However, it's essential to approach comparisons of prevalence trends across regions with caution due to variations in population characteristics among studies. Some studies focused on the general population, while others specifically examined obese or overweight children, potentially introducing differences in prevalence rates.

Numerous factors have been linked to the development of type 2 diabetes mellitus, including a family history of the condition, obesity, gender, sedentary lifestyle, and poor dietary habits. This aligns with the findings of Tanamas et al., who emphasized the substantial role of a family history of diabetes mellitus in its development [28]. They observed that parental diabetes and exposure to maternal diabetes during pregnancy are particularly influential "risk factors" for obesity and type 2 diabetes in children [28]. Among Native American children, the 10-year cumulative incidence of diabetes ranged from zero in those without parental diabetes to 8.2% to 16% among those with both parents affected by diabetes, depending on the age group studied [28-40].

Boone-Heinonen et al. highlighted that overweight/obesity resulting from compensatory infant feeding is a contributing factor in the development of type 2 diabetes mellitus [29]. Hypertension, dyslipidemia, and poor lipid profiles, often coexisting with obesity, have been widely established as factors associated with type 2 diabetes mellitus [28,30,31].

Notably, Lipton et al. found that obesity was common in individuals with type 1 diabetes mellitus, challenging the common perception that type 1 diabetes mellitus is more frequently associated with lean individuals or those with lower BMI, as also supported by Mottalib et al. [22,32]. The evidence of significant insulin resistance in the adipose tissues of obese individuals compared to lean counterparts, along with similar evidence in individuals with type 2 diabetes mellitus compared to those with normal glucose tolerance, suggests that increasing BMI and obesity play crucial roles in the progressive development of insulin resistance, ultimately leading to type 2 diabetes mellitus. This aligns with numerous publications describing obesity and overweight as implicated in the pathogenesis of type 2 diabetes mellitus through the pathway of insulin resistance [33,34]. A study by Santoro N. also noted that while adults may take a decade to transition into type 2 diabetes mellitus, the progression occurs twice as fast in obese young individuals, meaning they are more likely to develop type 2 diabetes mellitus within approximately five years [35].

Research conducted by Arslanian et al. revealed significant disparities in insulin sensitivity and β-cell function between youth and adults diagnosed with pre-diabetes and type 2 diabetes, potentially explaining the earlier onset of type 2 diabetes in specific young individuals compared to adults [36]. The study demonstrated that young individuals exhibit elevated insulin resistance levels and β-cells that are more responsive to stimulation than those of adults. This augmented insulin resistance may be influenced by the physiological insulin resistance associated with puberty, particularly among obese youth. Additionally, Arslanian et al. provided insights into the accelerated progression from normal glucose tolerance to type 2 diabetes mellitus in youth compared to adults [36]. This rapid progression leads to swift deterioration in β-cell function and reduced responsiveness to medications for lowering glucose levels [35-40].

Many of the reviewed studies utilized fasting plasma glucose and/or glycated hemoglobin (HbA1c) as diagnostic measures for diabetes mellitus. However, only a few incorporated 2-hour postprandial testing, which has been shown to diagnose more individuals with diabetes mellitus [37].

The majority of the included studies were cross-sectional in nature, which presents limitations in establishing a cause-and-effect relationship between childhood obesity and early-onset diabetes mellitus. To address this gap, further research using experimental designs, retrospective studies, and, to a lesser extent, prospective studies can be conducted to establish the temporal sequence from one disease entity to the eventual development of another. Additionally, it is crucial to differentiate between the different classes of diabetes mellitus, particularly between type 1 and type 2, which may require costly testing, such as autoantibody testing or estimating C-peptide levels, among other measures. As demonstrated by a few cohort studies in this review, larger population samples allow for results that are more representative of the broader population within a city, state, or region.

## Conclusions

In conclusion, the existing literature on childhood obesity and early-onset diabetes mellitus primarily originates from high-income countries, with limited representation from low- and middle-income countries. This disparity highlights a significant research gap in understanding the association between childhood obesity and early-onset diabetes mellitus on a global scale. Most studies reviewed fall into the categories of cross-sectional or cohort studies, offering valuable insights into prevalence and connections, but caution must be exercised when comparing prevalence trends across regions due to variations in study populations. Various factors, including family history, obesity, lifestyle, and dietary habits, have been linked to the development of type 2 diabetes mellitus in children. Furthermore, the review has shown the crucial role of obesity in the progressive development of insulin resistance, ultimately leading to type 2 diabetes mellitus, even in individuals with type 1 diabetes. To address the limitations in establishing causality and to differentiate between diabetes types, further research is needed, including experimental, retrospective, and prospective studies, as well as a focus on larger population samples. Additionally, incorporating 2-hour postprandial testing for diabetes diagnosis may provide a more comprehensive understanding of the disease.
